# A method for automatic segmentation and splitting of hyperspectral images of raspberry plants collected in field conditions

**DOI:** 10.1186/s13007-017-0226-y

**Published:** 2017-11-01

**Authors:** Dominic Williams, Avril Britten, Susan McCallum, Hamlyn Jones, Matt Aitkenhead, Alison Karley, Ken Loades, Ankush Prashar, Julie Graham

**Affiliations:** 1 James Hutton Limited, Invergowrie, Dundee, DD2 5DA UK; 2 James Hutton Institute, Invergowrie, Dundee, DD2 5DA UK; 3 Division of Plant Sciences, University of Dundee at the James Hutton Institute, Dundee, UK; 4 School of Agriculture and Environment, University of Western Australia, Perth, Australia; 5 School of Agriculture, Food and Rural Development, Newcastle University, Newcastle upon Tyne, NE1 7RU UK

**Keywords:** Hyperspectral imaging, Image segmentation, Field imaging, Raspberry, Phenotyping

## Abstract

Hyperspectral imaging is a technology that can be used to monitor plant responses to stress. Hyperspectral images have a full spectrum for each pixel in the image, 400–2500 nm in this case, giving detailed information about the spectral reflectance of the plant. Although this technology has been used in laboratory-based controlled lighting conditions for early detection of plant disease, the transfer of such technology to imaging plants in field conditions presents a number of challenges. These include problems caused by varying light levels and difficulties of separating the target plant from its background. Here we present an automated method that has been developed to segment raspberry plants from the background using a selected spectral ratio combined with edge detection. Graph theory was used to minimise a cost function to detect the continuous boundary between uninteresting plants and the area of interest. The method includes automatic detection of a known reflectance tile which was kept constantly within the field of view for all image scans. A method to split images containing rows of multiple raspberry plants into individual plants was also developed. Validation was carried out by comparison of plant height and density measurements with manually scored values. A reasonable correlation was found between these manual scores and measurements taken from the images (r^2^ = 0.75 for plant height). These preliminary steps are an essential requirement before detailed spectral analysis of the plants can be achieved.

## Background

Plant breeders are constantly striving to improve crop productivity through the breeding of plants with desirable agronomic traits that are also tolerant of abiotic stresses such as drought, mineral deficiency or heat stress and biotic stresses caused by pests and diseases. Raspberry is a perennial crop species grown widely in Europe and North America, and raspberry production faces a number of specific challenges. In terms of biotic stress, the spread of root rot disease caused by *Phytophthora rubi* has devastated many growers’ plantations and, once present, there is no effective treatment. The development of resistant raspberry varieties is therefore a key priority for breeders and a molecular markers breeding strategy is currently underway [1, 2]. Other challenges include combating insect pests, particularly root-feeding vine weevil larvae, and producing quality fruit [3, 4]. Recently, developmental disorders have also affected raspberry yields with increased incidence of crumbly fruit [4], lack of evenness and variable timings of bud break, and inconsistency in flowering and fruiting [5, 6]. Therefore, resilience in new variety development is essential to ensure future success in the raspberry industry.

Breeders therefore require tools for detecting the biochemical, physiological, and developmental responses to such stresses. These ‘phenotyping’ tools need to be capable of high-throughput screening of the huge number of plants that are typically generated and characterised as part of plant breeding programmes, whether for the mapping of quantitative trait loci (QTL) or for direct selection, and they need to be applicable in the field, rather than in controlled environments alone [7–9]. Sensors that detect reflected light from plants provide a technology that could satisfy these requirements. A wide range of sensors is now available for field use [10, 11]; these are most efficiently deployed on mobile field platforms [12, 13], while lighter sensors may also be deployed on unmanned aerial vehicles (UAVs) [14–16].

Among the many sensors available, there has been particular interest in the past few years in hyperspectral sensing of plant reflectance in the visible (400–700 nm), near infrared (NIR, 700–1000 nm) and the shortwave infrared (SWIR, 1000–2500 nm). This makes use of characteristic features of the reflectance spectrum that depend on biochemical or structural features of plant leaves and can be used for quantification of biochemical and physiological responses [17–19]. The power of hyperspectral remote sensing for detection and characterisation of plant responses to stress has been amply demonstrated by using in-field non-imaging spectroradiometry for the characterisation of traits as diverse as leaf biochemical composition [17], leaf water status [20] and responses to biotic stresses, however, the use of hyperspectral imagery has potential to greatly refine the power of this technique.

This study reports the development of an image processing method to support the application of a novel field phenotyping platform that incorporates two hyperspectral scanners: (1) SWIR scanner covering region 1000–2500 nm; and (2) a visible and near infra-red scanner (VNIR) covering the region 400–1000 nm with a combined capacity of recording 400 different wavelength bands. The scanners were mounted on a trolley pulled behind a tractor in such a way as to provide vertical scans of the lateral view of the plant canopy. Individual scans as the trolley moved forward were combined in a push-broom manner [21] to generate images of the lateral aspect of the plant row. A number of image analysis steps are required to extract the relevant data from the images for use in plant phenotyping.

Unfortunately, the shift to imagery as a phenotyping tool generates enormous amounts of data, necessitating the development of automated image analysis and statistical techniques for the required data reduction and synthesis [22, 23]. Image analysis techniques range from those that retain spatial information about the patterns of spectral variation (of particular interest for plant diseases or mineral disorders where there may be characteristic patterns of leaf colour [24, 25]) to the straightforward extraction of average spectra for the class of plant material of interest. The use of this latter approach is equivalent to the use of non-imaging spectrometers [26], but has the added power of eliminating error caused by those areas of the sensor field of view that do not correspond to the material of interest (i.e. plant leaf surfaces or even ‘sunlit’ leaf surfaces alone). Much previous work using spectral sensing for plant phenotyping has been conducted in highly controlled conditions [23], or focusses on relatively few wavelengths from red, green, blue (RGB) or multispectral sensors [27]. While growing plants in growth chambers or glasshouses allows environmental conditions to be closely controlled, it is important that phenotyping methods are applicable to the field conditions experienced by plants in a commercial setting. Imaging in field conditions produces additional challenges including the rapidly varying light exposure, influence of wind turbulence and the need to distinguish the target plants from a complex and varying background: in the field we are unable to move plants to an ideal position for imaging.

This study focusses on the critical step in image analysis of segmenting raw images to separate data linked to the leaf material of interest from that linked to other material such as stems, soil or other background features. Segmentation approaches for hyperspectral images can be split into two categories: those that attempt a pixel level classification based on the spectral signature of each pixel; and object based methods where location of the pixels is taken into account [28, 29]. Pixel based approaches have been commonly used for remote sensing data from either satellite or aircraft mounted scanners. These work well in situations with a variety of pixel classes that are well defined but where spatial resolution is poor. As the spatial resolution of hyperspectral imagers has improved, object based segmentation methods have become more common. These exploit information about the location of pixels in images to improve segmentation accuracy, detecting continuous objects that can then be allocated to a particular class. For field based systems, the limited number of potential object classes and high spatial resolution provides additional options for segmentation of image data.

The aim of this study was to develop an automatic method to segment the hyperspectral images identifying and labelling plants of interest and detecting reflectance standard. The approach chosen was to produce a single channel image from a combination of wavelength bands to segment important objects in the image. One major advantage of this approach is that dealing with only a few wavelength bands, reduces the image size and memory requirements for the image processing. It also allows generic machine vision techniques that are not specific to hyperspectral imaging to be used. The objectives were to overcome the main challenges associated with (1) detection of the white reference tile and (2) the accurate splitting of the image into individual plants.

## Methods

### Plants in the field

A replicated mapping population of 188 offspring with parents of Glen Moy and Latham raspberry varieties was planted at a field site at the James Hutton Institute, Dundee, UK. The advantage of using a mapping population for this work is that the image data can be related to molecular markers for traits via QTL analysis. Several linkage maps have been developed for traits of interest using this mapping population [2–5, 30, 31]. The plants were subject to two different biotic stresses (root rot and vine weevil, together with a stress-free control) and three different irrigation levels (control, drought, overwatered). The root rot plots were located on a site with known infection. Vine weevil plots were infested with eggs produced from live cultures held at the James Hutton Institute. Due to the extra space the need for biosecurity containment, the vine weevil plot only had two different irrigation levels. These treatments were combined to give eight different treatment combinations. Two replicates of each plant genotype were assigned to random positions within each block. The plots were uncovered in the field so control of water levels was limited by weather conditions. The plots were weeded and the raspberry plants were trained to grow on wires held up by wooden posts in line with standard practice for growing raspberry.

### Imaging set up

**Fig. 1:**
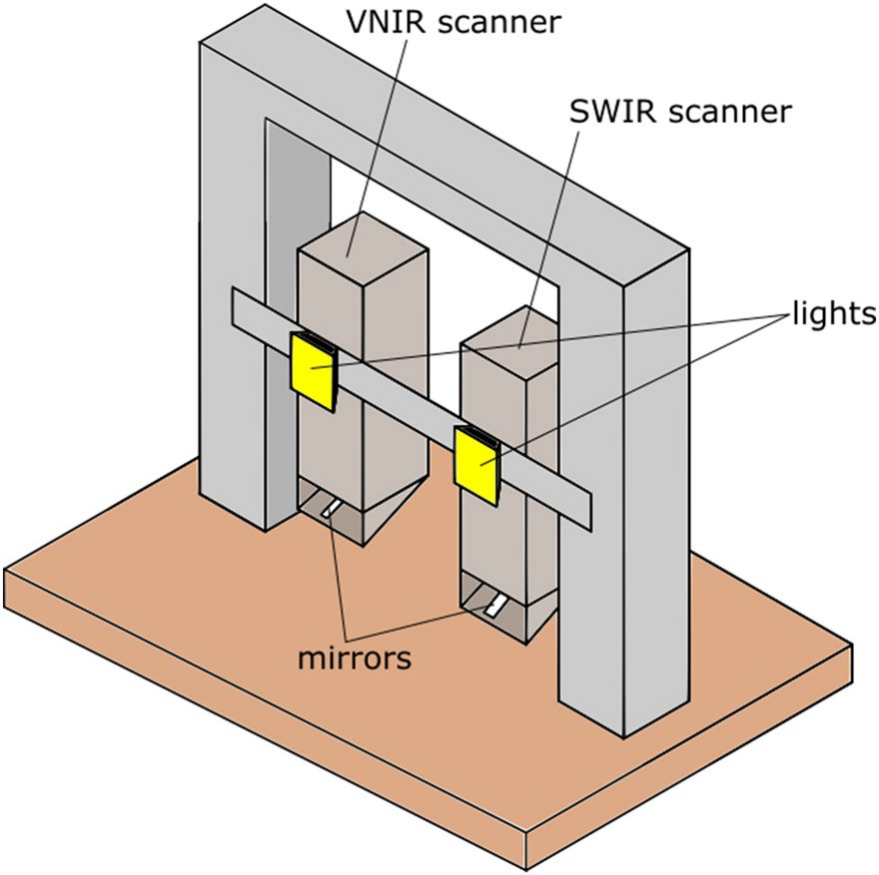
View of scanner set up, wires, power supply and frame to hold background are not shown for clarity

**Fig. 2:**
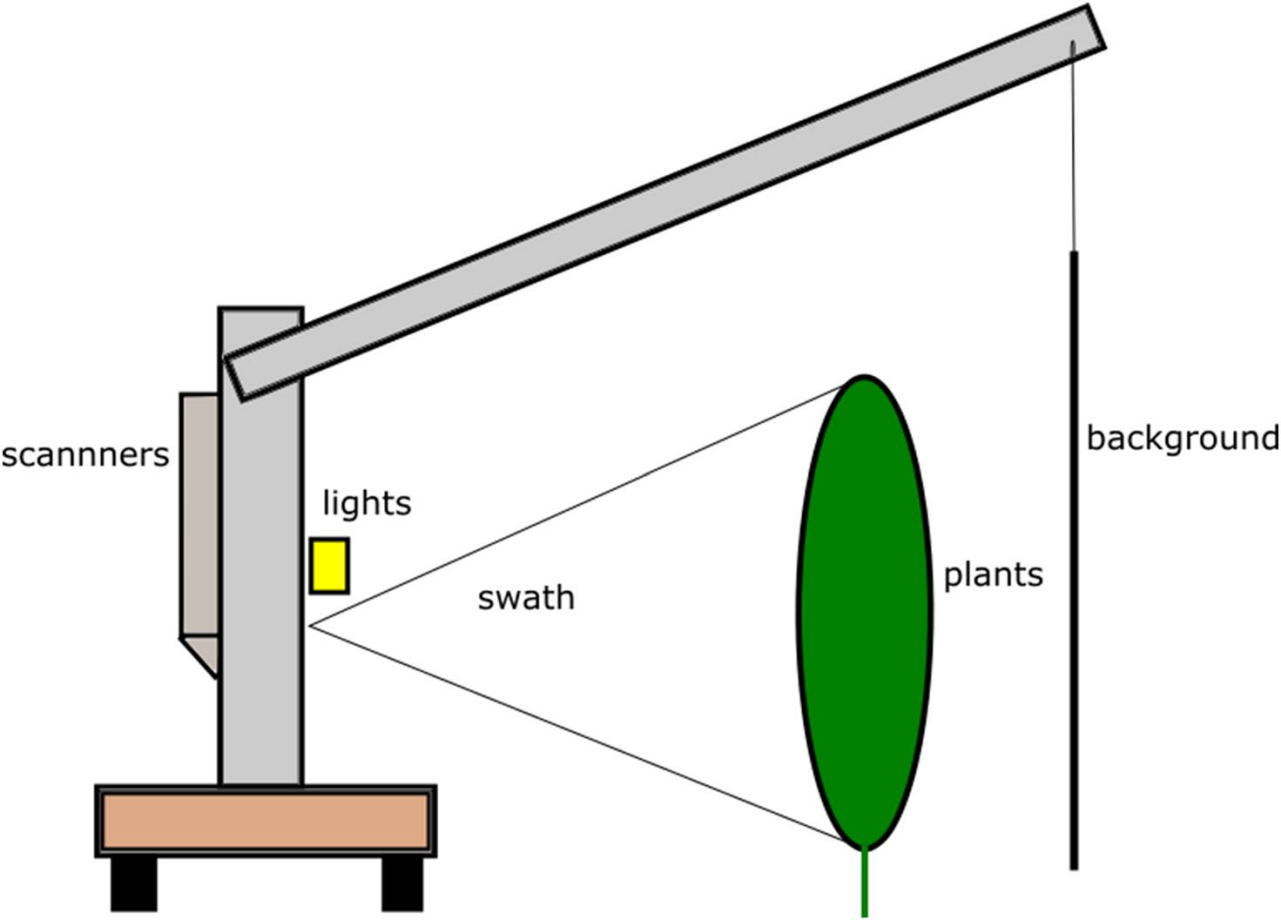
Rear view of platform showing target plants

The images were acquired using two scanners (see Fig. 1). Both of these scanners worked on a pushbroom line scanning method acquiring a single vertical line of an image at a time. The scanners were moved horizontally to generate a 2D hyperspectral image comprising multiple vertical image scans. The VNIR scanner was manufactured by Gilden Photonics, and acquired images in the wavelength range of 400–896 nm. Binning (4×) was carried out during the imaging process which produced 178 wavelength bands in the 400–896 nm range and 402 vertical pixels covering a swath of approx. 1.2 m at the target distance. The SWIR scanner was manufactured by Specim, with a wavelength range of 895–2506 nm. This scanner was used at full resolution giving 278 wavelength bands within that region and 378 vertical pixels covering a swath of 1.2 m at the target range. To discriminate target plants in the row from plants in rows behind the target array, we suspended a cardboard sheet behind the plants (see Fig. 2). A spectral reference tile was attached to this cardboard sheet so that it was continuously in the scanner field of view, thus allowing calibration against changing light levels. Two 400 W RS components halogen floodlights were mounted on the platform just above the scanners to provide illumination at similar orientation to the scanners; these lights were used at all times, and allowed imaging to proceed in cloudy conditions when natural irradiance in the short wave infrared region is very low. Adding in artificial lights does add complexity to the spectrum detected as the spectra become a mixture of natural and artificial light. In bands with high atmospheric absorbance, light will mainly be from artificial lights.

The SWIR and VNIR scanners were mounted at the same height with a 30 cm horizontal offset between them. They were oriented to cover the same area of the studied plants. The images from each scanner were analysed independently rather than attempting a registration of the two images. Registration was not attempted as it would add in an extra source of potential error to the data analysis. A slightly differing vertical resolution would mean any registered results would need to be interpolated. The horizontal offset between the two scanners meant there was a time delay between imaging the same plant by the two different scanners adding in potential error on any days with sufficient wind speed to disturb the plants.

### Imaging protocol

**Fig. 3:**
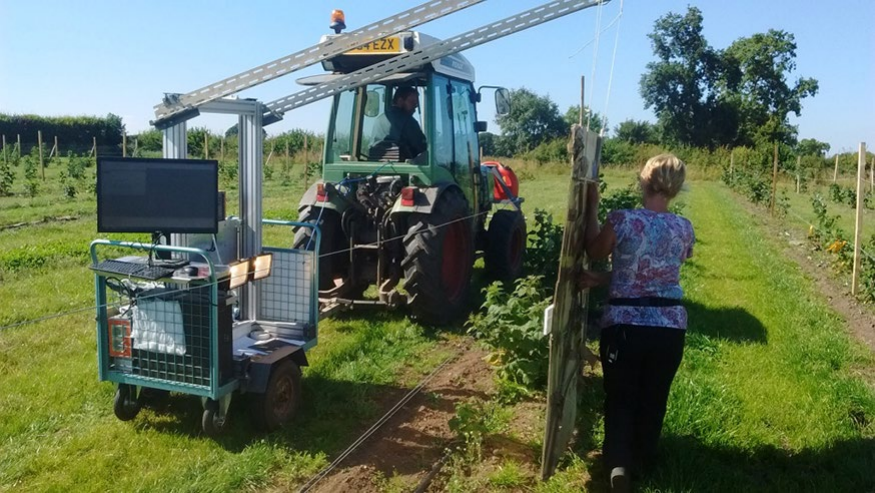
Photo of imaging platform in action

The plants were imaged in 50 m rows of 48 plants every 2 weeks throughout the 2016 growing season (May–September). At the start of each row, the white reference tile was imaged without any plants in the image. The exposure of the scanners was then adjusted so that the white reference produced values between 50 and 90% maximum intensity of the scanner to avoid over or under exposure in the images. The reflectance of the white reference was much higher than that of plants in the visible region where light intensity is highest, so any problems with over exposure were encountered on the reference tile rather than the plants. Once the exposure had been set, the scanners were all switched on and the tractor towed the imaging platform (Fig. 3) down the row at a constant velocity of 2.7 m s^−1^, with the cardboard background held stable by a person walking alongside. The exposure was rechecked and adjusted at the start of each row of imaging.

### Image preprocessing

Dark current was removed from the SWIR images by subtracting the spectrum acquired when no light was reaching the detector from the spectrum returned in each image. The dark current spectrum is unique to each vertical pixel within the image. The dark current for the VNIR scanner was much smaller and removal was not necessary to achieve good quality images.

The imaging method (described above) ensured that all plants of interest were contained in the images. The first step of image processing involved manually cropping the ends of each image to remove non-target image data, which required the user to click on the image, at points indicating the start and end of the row of plants. A three band true colour representation of images was used for this purpose. No further manual intervention was required for image processing.

Further processing of image data differed slightly between the VNIR and SWIR scanners.

### Automatic plant detection

#### VNIR

**Fig. 4:**
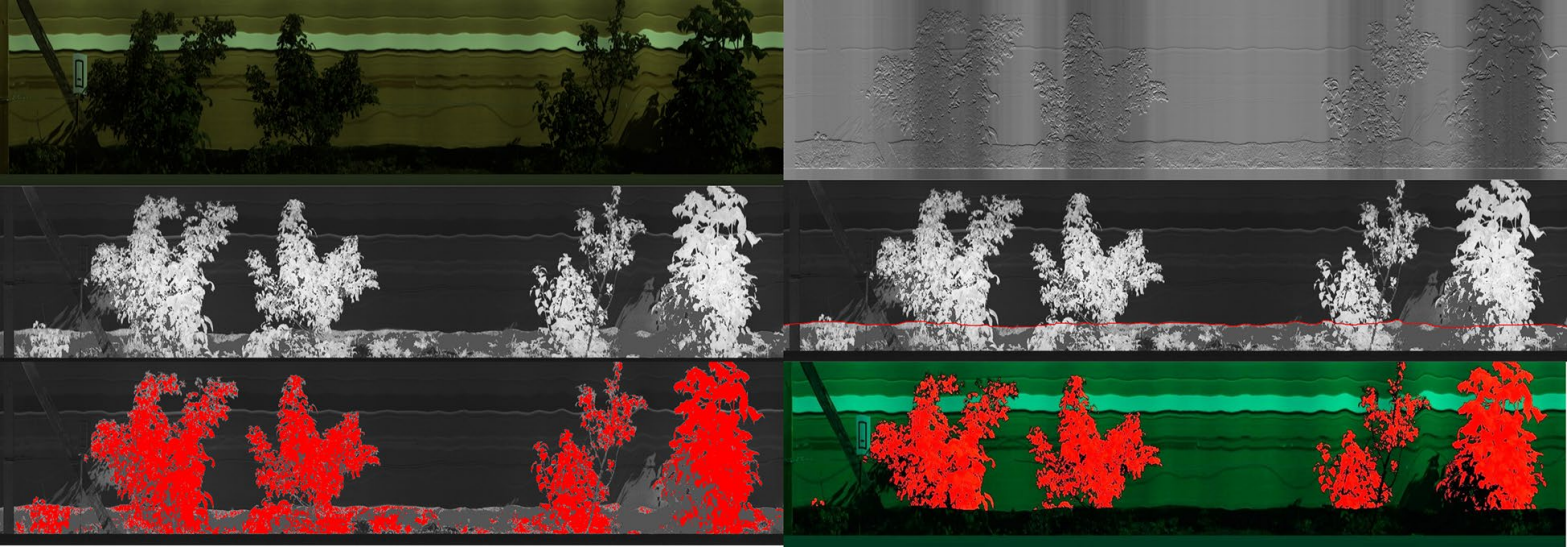
Example image showing segmentation steps for VNIR data. Top left is true colour representation of data. Centre left shows NDVI image. Bottom left shows segmented plant location marked in red on NDVI image. The top right is the cost function that is minimised to find the bottom boundary. Centre right is the boundary marked on NDVI image and bottom right is final segmentation of plants with grass removed marked on original true colour image

Discrimination of plant leaf material from background pixels is a relatively simple task in the visible and near infra-red region. A fixed threshold was applied to the normalised difference vegetation index (NDVI) values [32] based on visual inspection of a number of images. NDVI was defined as NDVI = (IR − red)/(IR + red) where red was defined as the mean intensity between the wavelengths, 650 and 680 nm, and IR as the mean intensity between the wavelengths, 710 and 740 nm. An example NDVI image can be seen in Fig. 4. After applying the NDVI threshold, the image was eroded using a 3 × 3 cross shaped structural element [33]. This has the effect of removing any pixel from the detected plant if one of its immediate neighbours has not been classified as a plant. This removes the few stray pixel detections and any mixed pixels encountered on the edge of leaves.

The space between the rows of raspberry plants was grassed, which means that grass appeared at the bottom of each of the images. Our initial segmentation included this grass as part of the plant. An approach was developed to remove this non-target plant material by detecting the bottom of the cardboard background. Graph theory was used to find an edge across the NDVI image. Graph based segmentation has been used to solve a number of different image segmentation problems [34], including both plant segmentation problems [35] and much wider applications [36, 37]. The main advantages of this technique are the low computational power required and the lack of need for training data.

Graph theory segmentation works by using a cost function for each pixel then finding the shortest path across the image to minimise this function. The cost function used here was:

$${\text{C}} = {\text{e}}^{{ - {\text{I}}({\text{x}})}} ( 1- {\text{g}}_{\text{y}} )$$

where g_y_ is the vertical gradient of the NDVI image, I(x) is the mean intensity of a vertical slice of the image at point x and C is the cost function to be minimised [34]. The additional brightness penalty was added to reduce the effect of the cost penalty in the situations where there are plants which hide the boundary between the cardboard and grass. Where the cost penalty is zero, a straight horizontal path would be produced.

The cost function was minimised using the following expression

$$t(x,y) = \left\{ {\begin{array}{*{20}l} \infty & {y < 1,y > m} \\ {C(x,y)} & {x = 1} \\ {\mathop {\min }\limits_{{}} \left( \begin{aligned} & t(x - 1,y - 1) + C(x,y) + \lambda _{{ver}} , \\ & t(x - 1,y) + C(x,y), \\ & t(x - 1,y + 1) + C(x,y) + \lambda _{{ver}} \\ \end{aligned} \right)} & {otherwise} \\ \end{array} } \right.$$

where t(x, y) is the cost to reach point (x, y), C(x, y) is the cost function giving cost at point (x, y), x is the horizontal direction index and y is the vertical direction index and m is the image height. $\lambda_{ver}$ is a vertical cost penalty added to favour selection of lines with limited vertical movement.

The area below the boundary found was removed from detected plant material.

#### SWIR

**Fig. 5:**
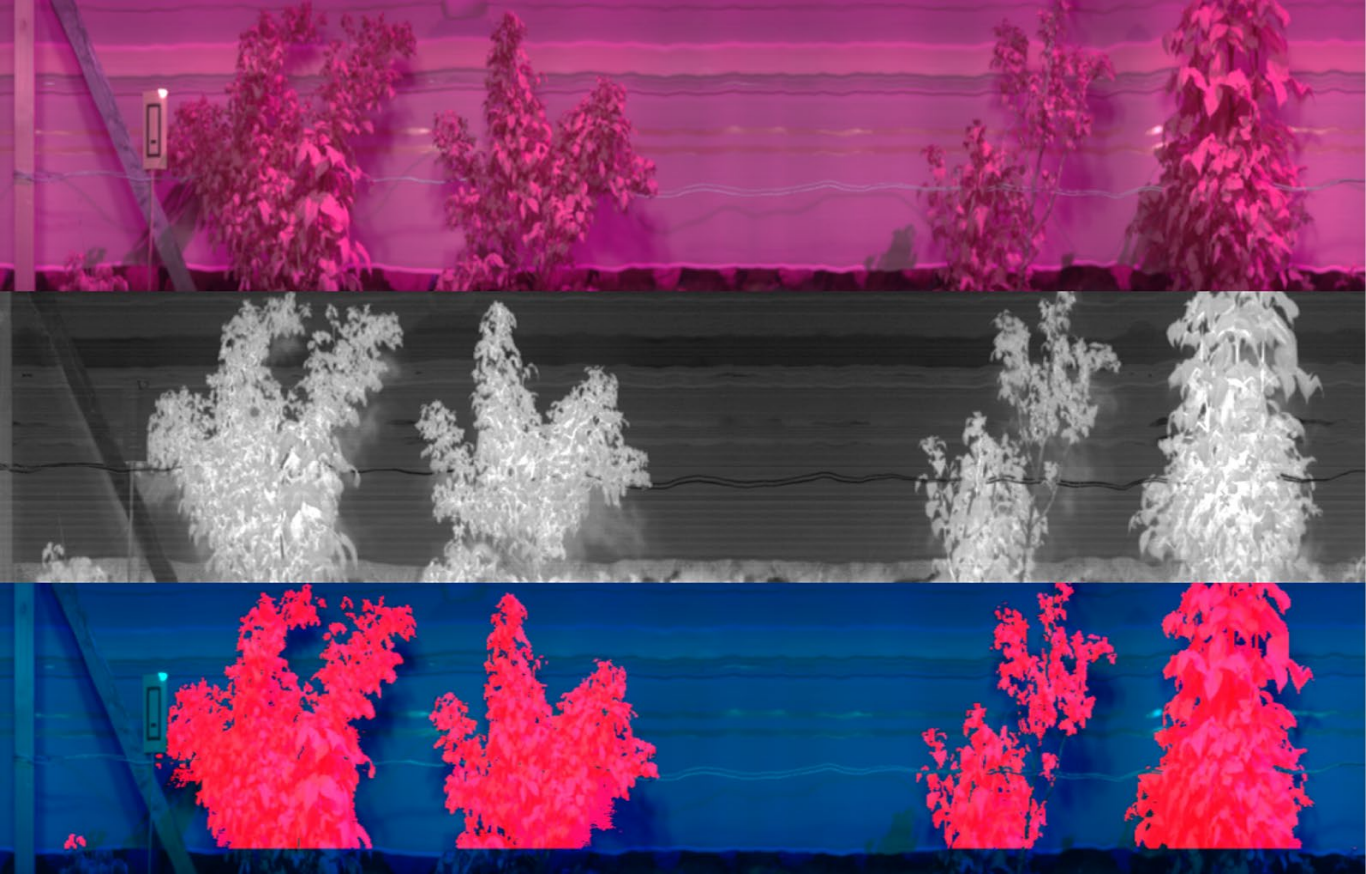
Example imaging showing SWIR image segmentation. Top image is false colour image of SWIR data. Centre image is a normalised difference image used to detect plants, where it is clear that plants can be clearly distinguished from the background, although a fainter signal of their shadows can also be seen. Bottom image shows plant segmentation marked on original false colour image within which the bottom section was cropped off

Discrimination of plant material is more challenging in the SWIR region of the spectrum. A method that exploits the different shape of the water absorption regions of the spectrum in the plant and background was developed. A normalised difference ratio between the intensity at 1375 and 1411 nm was found to discriminate between plant and background. A grayscale image, see Fig. 5, was produced using the following equation:

$${\text{Ndif}} = \left( {{\text{I}}_{ 1 3 7 5} - {\text{I}}_{ 1 4 1 1} } \right)/\left( {{\text{I}}_{ 1 3 7 5} + {\text{I}}_{ 1 4 1 1} } \right)$$

where I_1375_ is the mean intensity of the image for 3 bands centred at 1375 nm and I_1411_ is the mean intensity of the image for 3 bands centred at 1411 nm. Otsu’s method [38] was applied to find a threshold to best segment the image.

The problem of the removal of grass from the foreground is also presents itself in the SWIR images. Here a different approach was used because attempts to replicate the method used in the VNIR images did not produce good results, due to noise in the SWIR images with a horizontal consistency that persisted even after dark current removal; this noise created false edges that were picked up frequently. Instead, a fixed part of the bottom of the image was removed. Although this resulted in removal of a larger section of the image than the VNIR method, the approach ensured the grass was reliably removed.

### Automatic detection of white reference

A method was developed for automatic detection of the white reference tile in each image to allow correction of changes in light levels between images.

#### VNIR

For detection of the white reference, the mean intensity of the spectrum between 455 and 480 nm was calculated. This region of the spectrum was chosen as a bright area, with best contrast between the white reference and surrounding objects. The white reference was continuously in the field of view although it was obscured behind posts and plants for part of the images. The same method that was used to detect the bottom of the cardboard background was applied (described above). First, the bottom of the white reference was found using graph theory, with the same cost function as earlier, but without the intensity adjustment:

$${\text{C}} = 1- {\text{g}}_{\text{y}}$$

where g_y_ is the vertical gradient of the mean intensity of the image and C is the cost function to be minimised. The same minimisation technique was used as described previously. In order to detect the top edge of the white reference, a shape function (d) was generated from the bottom of the white reference. This used an expected width for the white reference to favour detection of an edge at a known distance from the bottom edge. This shape function was included to give the following cost function:

$${\text{C}} = 1- {\text{g}}_{\text{y}} + \lambda_{\text{dist}} \times {\text{d}}$$

where g_y_ is the vertical gradient of the mean intensity image, λ_dist_ is the distance weighting term, d is the shape function and C is the cost function to be minimised. This cost function was then minimised to find the lowest weighted path across the image.

Following the initial detection of the white reference between the two bounding lines, further image corrections were carried out. In principle, the white reference was continuous across the image. In reality, the white reference was occluded in parts of the image by plant material and wooden posts.

**Fig. 6:**
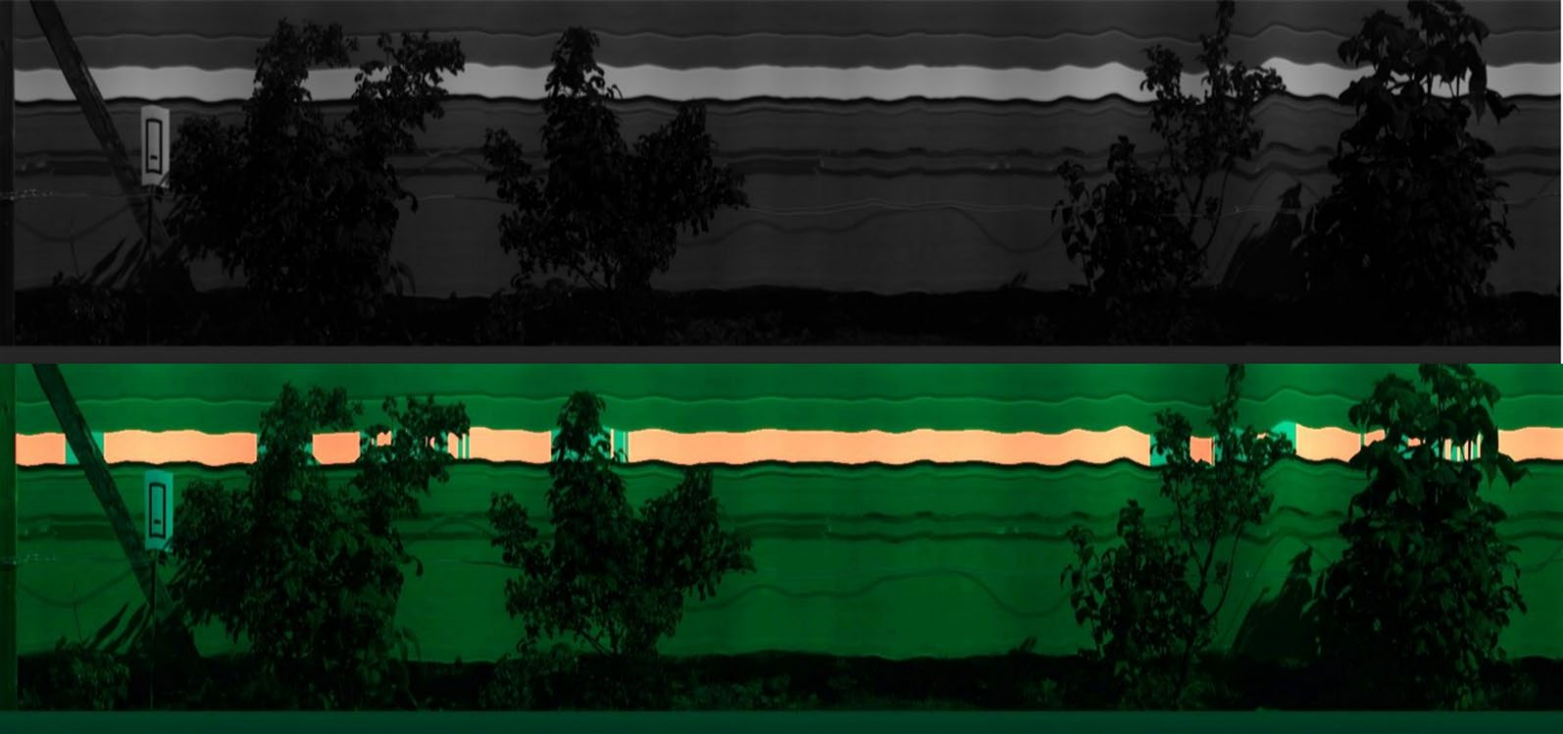
Example image showing white reference detection in VNIR images. Top image shows grayscale image of the mean intensity between 455 and 480 nm and bottom image shows the detected white reference marked in red

Removal of these objects from the image was based on the fact that they were darker than the white reference. The variance of each vertical scan of the white spectrum was calculated. Any columns with variance twice the mean variance were removed, and the mean intensity of each column remaining was calculated. A threshold was then applied to remove the darkest columns, which most likely contained image data from leaves or wooden posts. This generated a mask that marked the locations of the white reference within the image. This method is shown in Fig. 6.

#### SWIR

**Fig. 7:**
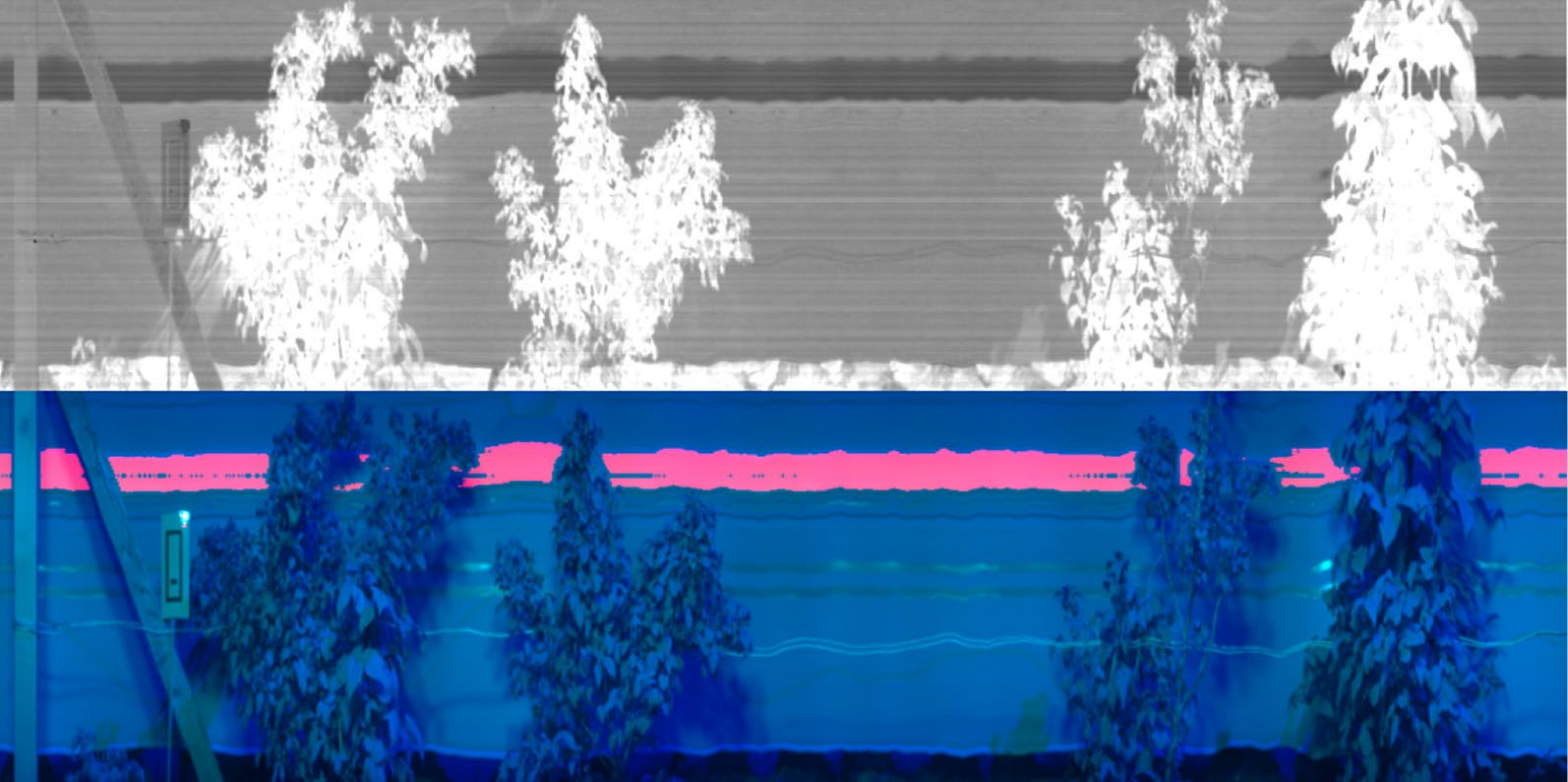
Example image showing white reference detection. Top image shows the normalised difference between 1620 and 1537 nm used to detect white reference, which can be seen as darker pixels in image. Bottom image shows the detected white reference marked on false colour image used previously in Fig. 5

Due to the confounding issues caused by noise in SWIR images, an alternative method based on finding a suitable threshold was used to detect the reference. The first step was to find suitable wavelengths that separated the white reference from the rest of the image. Inspection of the images revealed, a normalised difference of the mean intensity of three bands centred at 1620 and 1537 nm was able to differentiate the white reference (Fig. 7).

**Fig. 8:**
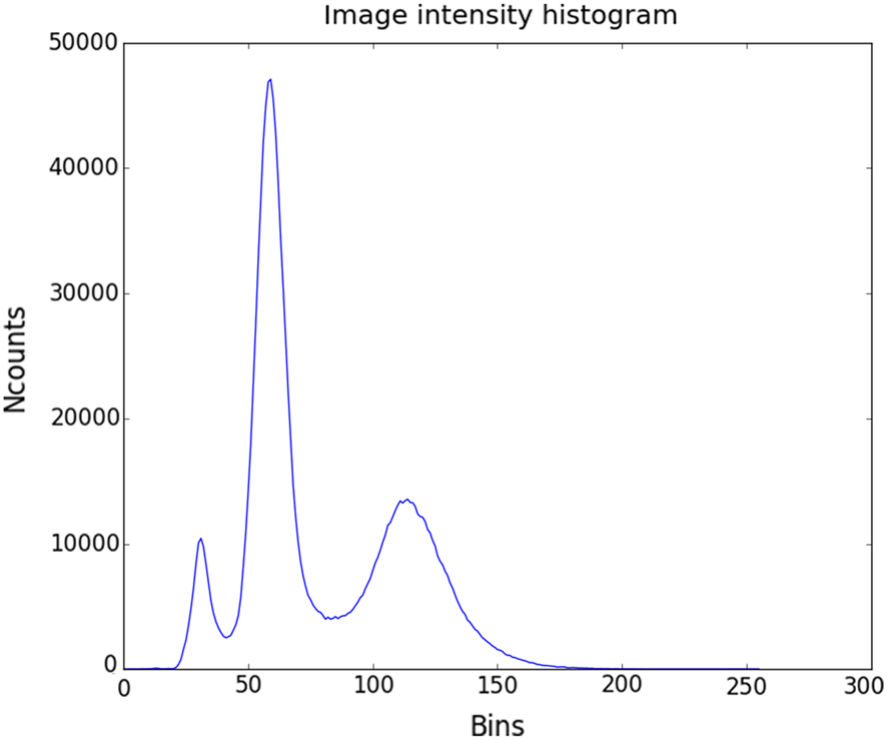
Histogram of image intensity for normalised difference image used to detect white reference. Peak on left corresponds to pixels belonging to white reference, peak in centre to pixels that are part of the back ground and peak on right to pixels mostly belonging to plants

A histogram of intensity (Fig. 8) indicates three reasonably well defined regions, corresponding to the white reference, plants and background (respectively). The first peak corresponds to the white reference, in order to segment this, a local minimum was found, using the point that included 10% of all pixels as a starting point. The value of the intensity at this point was used as a threshold to segment the image. The image was morphologically eroded using a cross structuring element to remove stray pixels and small objects that were incorrectly classified as white reference.

### Splitting into individual plants

Individual plants in each row of the trial were separated by a distance of 1 m (48 plants per row), which is larger than commercial plantations, but facilitated the task of splitting image files into individual plants. Separating individual plants was necessary as each plant in the row of an image was a unique genotype in the mapping population. Since the image was collected at a constant speed, the simplest approach would be to split each image into 48 equal sized sections, with each containing a separate plant. In practice, this approach was not robust due to errors caused by variation in tractor speed or plant growth that deviated from vertical, so a method based on our prior detection of plant material was used instead.

The number of pixels assigned as ‘plant’ material for each column was calculated. Peaks in this should correspond to plants and minima should correspond to gaps between plants. A peak finding function was used to identify peaks, while enforcing a minimum distance of 80% of the expected plant separation. The points midway between each peak were used to split the image into individual plants. Any areas where plant separation was too large were split into multiple regions to account for any plants that had been missed by the peak finding method. The number of plants identified and the known number of plants in the image was matched by either merging small regions or splitting large regions. Finally, the splitting points were adjusted to identify a local minimum, in the number of plant pixels, corresponding to an optimum point for splitting the image.

**Fig. 9:**
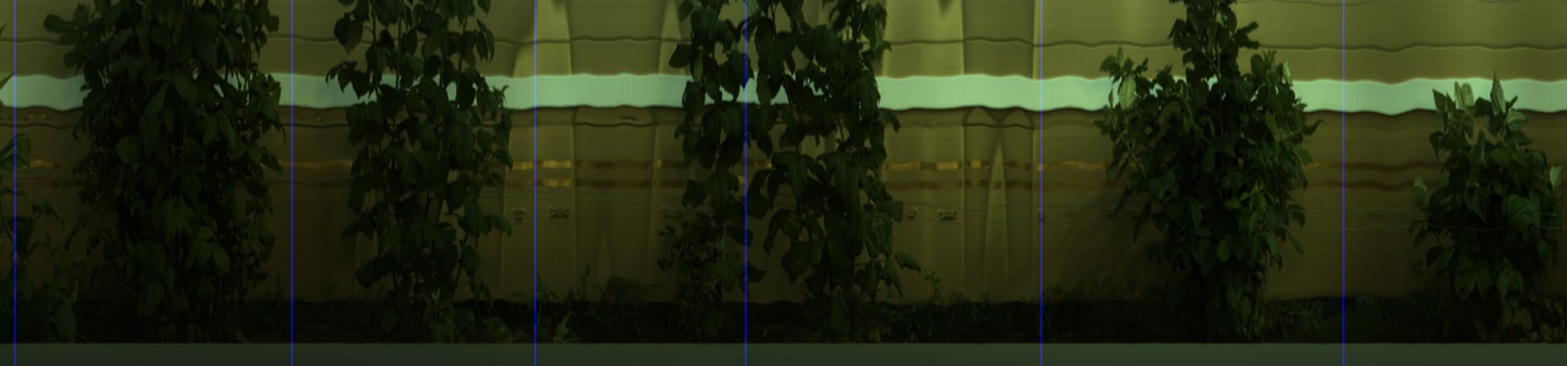
Example image segmented into plant sections. Small areas of overlapping plant can be seen in centre of image that cross the lines. These are removed by excluding a 10% boundary on either side of the marked lines from the segmentation

This method did not produce a perfect splitting of plants, but ensured that image splitting was as accurate as possible. In some cases, there was a degree of overlap between adjacent plants in the image, with branches of one bush intermingling with that of the neighbouring plant, indicating that improved plant management and control is important to accurately split the images. In order to reduce the effect of this overlap, for each detected plant, 10% of the plant width was cropped off each side to exclude areas where two plants overlapped (see Fig. 9 for an example).

This method was applied to both VNIR and SWIR images.

### Extracting data

Using the segmented image data, the spectrum of the white reference tile was used to normalise the image from pixel intensity values to reflectance. The image was divided into sections of fixed size then dividing the spectrum of each pixel in that section by the mean white reference spectrum from that section.

Finally, the normalised reflectance spectrum of each plant was extracted, together with measures of the plant height and mean density of the plant, using the binary segmented plant image. These final two measures were used to evaluate performance of the segmentation by comparison with visual scoring of the plants in each image for height, density and diameter.

## Results and discussion

**Fig. 10:**
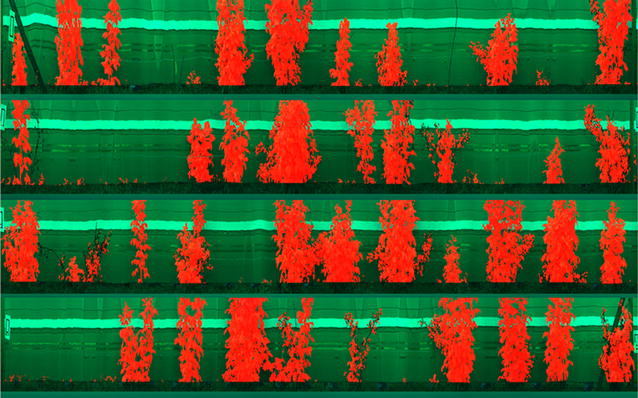
Example image with segmentation results marked on it. Each row should contain 12 plants but some plants are missing

The study aimed to produce an accurate segmentation of individual plants from a hyperspectral image. This was achieved by developing a method to detect the white reference tile for normalising the collected data and a method to split the image into individual plants. Accurate validation of the segmentation method is a challenging task. Although visual inspection of the results (example image in Fig. 10) showed good agreement between the segmentation results and the plants quantitative validation of the results is more difficult because replicating the segmentation by hand would be time consuming and prone to errors. As an alternative validation method, manual observers classified the plants visually for plant height and cane density. These scores were compared with the measurements of plant height and density obtained from the segmented images. The comparison was carried out over a total of 44 images containing 1440 different plants.

**Fig. 11:**
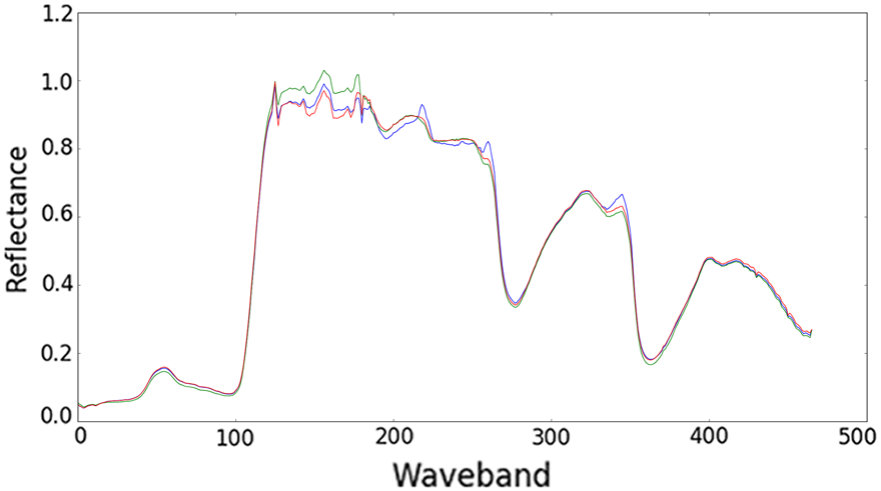
Example mean spectrum of three plants. Data from two cameras has been combined after image processing to give mean spectrum of plant spectrum covering both VNIR and SWIR regions

An example of the mean spectrum generated from three neighbouring plants is shown in Fig. 11. This shows we are able to generate meaningful spectrum for plants using the method described here.

### Comparison of height measures

**Fig. 12:**
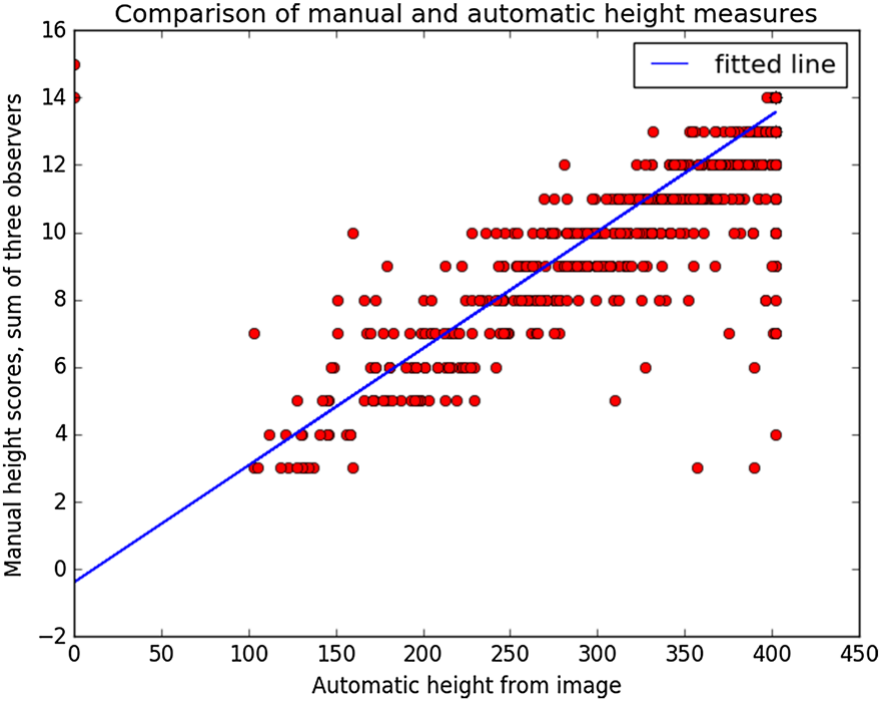
Relation between plant height values obtained by automated segmentation of image data and visual scores assigned by manual observation. The fitted line has equation y = − 0.395 + 0.0347 × x (r^2^ = 0.75)

The sum of the three manual observer’s height scores was compared with the values generated from images by the automated segmentation method (Fig. 12), and showed good correlation with an r^2^ value of 0.75. The extreme values in top left and bottom right corners are probably evidence of disagreement in plant labelling between manual and automatic measures.

**Fig. 13:**
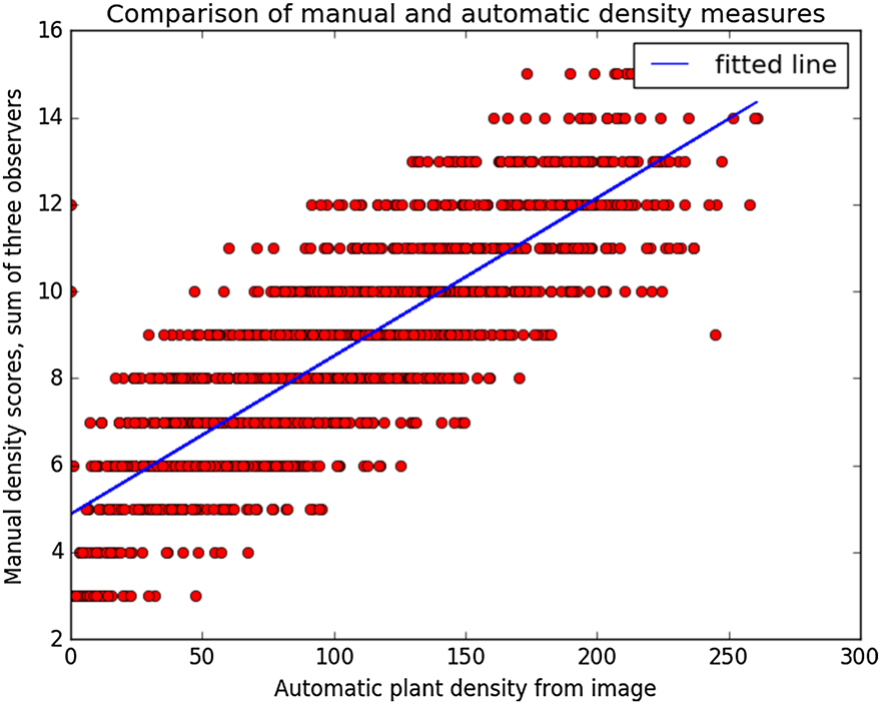
Relation between plant density values obtained by automated segmentation of image data and visual scores assigned by manual observation. The fitted line has equation y = 4.87 + 0.0364 × x (r^2^ = 0.68)

Similarly, manual measurements of cane density showed a positive relation with mean number of plant pixels per plant obtained from image segmentation (Fig. 13; r^2^ = 0.68). The latter value is not directly equivalent to plant density scores. There were a number of outliers where the automated processing failed to locate a plant that was detected by the manual scoring, showing there are improvements that could be made to the automated segmentation method for splitting plants in the image.

Although the main purpose of the imaging platform was not to collect plant height and density data specifically, the comparison of manual measurements with automated measurements for height and plant density provided a means of validating the methods used to process image data collected using the platform. Future work will focus on the spectrum produced by imaging. This requires an accurate segmentation method to ensure that image data provides information about the spectral response of the target plants, rather than the background or neighbouring plants. Establishing the automated image processing methods for segmenting and splitting the image data will facilitate the analysis of the plant reflectance spectrum and responses to a range of biotic and abiotic conditions.

## Conclusion

The study aim and objectives were achieved by developing a method for the automatic segmentation of target plant material in hyperspectral images of raspberry plants and the splitting of these images into individual plants. This is the first time that the segmentation of ground based hyperspectral images of bush crops has been attempted. Segmentation was carried out on images generated from a normalised difference between particular bands. Novel bands were selected for the segmentation of SWIR images. The segmentation was carried out using thresholds and graph theory. A small amount of manual intervention was required as an initial step to remove irrelevant sections at the start and end of images.

The performance of this technique has been partially validated by comparison of manual and automated measures of plant shape. The good correlation between manual and automated measurements confirmed the value of our segmentation method. The evaluation of segmentation performance was carried out in this way, as manual segmentation of the images would have being labour-intensive and also subject to observer bias. Manual inspection of segmented images indicated good quality segmentation in all images.

This method has been developed to solve a particular problem encountered with imaging perennial plants in field plantations. Challenges of irregular plant growth and inconsistent lighting had to be overcome to enable the segmentation to succeed. Due to the relative novelty of using ground based hyperspectral scanners in field environments, there are no widely applicable segmentation tools available at present. The methods described here have been developed with a particular imaging set-up in mind, although using details of the approach, other researchers may be able to alter and adapt the methods to solve their own image segmentation challenges.

This study reports development of the imaging platform and associated imaging processing methods. In future, we aim to implement these methods in further work to analyse the utility of the extracted image data for a range of applications.
